# The ethnic differences of stroke in Yakutia

**DOI:** 10.3402/ijch.v72i0.21221

**Published:** 2013-08-05

**Authors:** Sargylana A. Chugunova, Tatiana Ya. Nikolaeva

**Affiliations:** 1 Yakut Science Center of Complex Medical Problems of the Siberian Branch of the Russian Academy of Medical Sciences, Yakutsk, Russia; 2 North-Eastern Federal University named after M.K. Ammosov, Yakutsk, Russia

**Keywords:** epidemiology, ischemic stroke, haemorrhagic stroke, incidence, ethnic differences

## Abstract

**Background:**

In Yakutia, the morbidity and mortality from stroke increased in the past 2 decades. Stroke share in the total mortality structure increased significantly. According to the autopsies, haemorrhagic stroke (HS) was more common in indigenous patients.

**Objective:**

The aim of the study was to examine ethnic features of stroke patients of indigenous and non-indigenous ethnicity admitted to Regional Vascular Center (RVC), Yakutsk.

**Design:**

The study used data from a hospital stroke registry, which took into account the cases of acute stroke in 2011. Stroke type and aetiology were determined by clinical examination, computed tomography and magnetic resonance imaging studies, cerebral angiography and ultrasound of cerebral vessels.

**Results:**

A total of 1,108 patients were hospitalized (51.4% male, n=569) in 2011. The mean age was 60.5±12.9 years, male: 59.1±12.8, female: 61.9±13.05. Five hundred and ninety-two ischemic strokes (IS; 53.4%), 236 HS (21.3%), 280 transient ischemic attacks (TIA; 25.3%) were diagnosed. Patients who had a stroke were divided into 3 groups according to their ethnicity: native (n=411; 49.6%), Russians (n=347; 41.9%) and other nationalities (n=70; 8.5%). When comparing the incidence of HS in different ethnic groups, it was found that indigenous patients had more cases of HS than Russians (38% vs. 20.2%, p<0.05; adjusted odds ratio=2.42; 95% confidence interval: 1.72–3.41). Mean age of IS and HS indigenous patients had no significant differences compared with the average age of Russian ethnicity patients (p=0.69; p=0.201, respectively).

**Conclusions:**

The data from this study suggest that among the patients who suffered from stroke in the indigenous population, the share of a haemorrhagic form washigher than those of non-indigenous Caucasians. At the same time, the average age of patients, both having IS and HS had no significant differences by ethnicity. Further studies are needed to establish the causes of ethnic differences of stroke in Yakutia.

Cerebral stroke is the second leading cause of death in Russia (1). In Yakutia, the morbidity and mortality from stroke increased in the past 2 decades. For example, the stroke rate increased from 244.1 per 100,000 in 2006 to 295.9 in 2010 (2). Stroke share in the total mortality structure increased significantly (approximately double in the past 20 years). The increase was mainly due to the haemorrhagic form of stroke. According to the autopsies, haemorrhagic stroke (HS) was more common in indigenous patients (3,4).

One-fifth of Yakutia is occupied in Russia’s territory and 40% of Yakutia’s territory is located beyond the Arctic Circle. The overall population of this region is more than 950,000 with the adult population comprising more than 730,000 (5). Yakutia is home to 2 major ethnic groups that make up 94.1% of the population:

- the indigenous, relating to the Mongoloid (Asian) race: (Yakuts (49.9%), Evenks (2.2%), Evens (1.6%), Dolgans (0.2%), Yukagirs (0.1%) and Chukchi (0.1%).
- the non-indigenous, relating to the Caucasians Russians (37.8%) and Ukrainians (2.2%) (5).

## Objective

The aim of this study was to examine ethnic features of stroke patients of the indigenous and non-indigenous ethnicity admitted to the Regional Vascular Center (RVC), Yakutsk.

## Design

The study used data from a hospital stroke registry, which took into account the cases of acute stroke in 2011. The patients admitted to the stroke unit at the Department of Neurology of the Regional Vascular Centre (RVS, Yakutsk) in 2011 were consecutively included in our study. All patients with an acute cerebrovascular accident, without exception, are admitted to the RVS from Yakutsk city, with a population of 269,601 inhabitants (5). These were patients with acute symptoms of cerebral circulation impairment in the past 24 hours and/or fluctuation or progression for a long time (6). Patients from other parts of the region with indications, which include the need for high-tech stroke treatment methods, are hospitalized in the RVS.

A trained neurologist diagnosed signs of acute cerebral circulation impairment. Type and aetiology of stroke were determined by clinical examination, computed tomography (CT; 64-slice) and/or magnetic resonance imaging (the magnetic field strength 1.5 T) of the brain, Doppler and duplex sonography of cerebral and brachiocephalic vessels, and cerebral angiography. Neuroimaging data were studied and described by a CT doctor.

Patients with diseases classified by codes I 60–I 64, G 45–G 46 to International Statistical Classification of Diseases and Related Health (10th revision) were included in this study (7,8). Separation by stroke type was conducted as follows: cerebral infarction was considered as a type of “ischemic stroke” (IS), intracerebral haemorrhages, subarachnoid haemorrhages, subarachnoid–parenchymal haemorrhages, isolated intraventricular haemorrhages of non-traumatic aetiology were typed as “HS”.

To achieve the research objective, the following characteristics were analyzed: age, gender and type of stroke in indigenous and non-indigenous patients.

The patients were separated based on ethnicity: Mongoloid (Asian) race representatives (Yakuts, Evens, Evenki, Dolgans, Yukagirs, Chukchi) were considered as “the indigenous”, Caucasians (Russians, Ukrainians) – “the non-indigenous”. The latter group subsequently is called “the Russians”. Members of other non-indigenous ethnicities (Buryats, Tatars, Kyrgyz, etc.) made up the group of “the other”.

A total of 1,108 patients in total were screened with a diagnosis of “acute cerebrovascular accident”, including a randomized group of 828 patients with a diagnosis of “acute stroke”.

## Statistical analysis

The analysis was performed using the software package STATISTICA version 6.0.

Checking of the normal distribution of quantitative signs was performed using the Kolmogorov–Smirnov test. For all quantitative signs in the compared groups, arithmetical mean and standard deviation was evaluated. These descriptive statistics in the text are presented as M±σ, where M is the mean and σ is the standard deviation. Quantitative signs that did not have a normal distribution were described by medians (Me) and quartiles [Q1; Q3].

For quantitative traits with normal distribution, a t-test was used. For comparison of the parameters of the 2 groups that did not have a normal distribution, a non-parametric method Mann–Whitney U-test was used.

The study of the relationship between pairs of discrete qualitative traits was performed using analysis of paired contingency tables. Besides the evaluations Fisher’s exact test and the achieved level of statistical significance, strength of associations in the values of the relative risk (OR) with 95% confidence interval (CI) was assessed.

## Results

A total of 1,108 patients with acute cerebral circulation impairment were examined (males: n=569; 51.4%). The average age was 60.5±12.9 (range: 16–101 years) in the general group: male−59.1±12.8 (range: 16–88 years), female−61.9±13.05 (range: 21–101 years). Thus, the average age of male patients was lower than that of female patients (p=0.0003 [t-test]).

The general group was made up of: indigenous patients (n=570; 51.4%), Russians (n=440; 39.7%) and those of other ethnic groups (n=98; 8.8%).

Five hundred and ninety-two IS (53.4%), 236 HS (21.3%) and 280 (25.3%) transient ischemic attacks (TIA) were diagnosed.

In the group of indigenous patients, there were 255 IS (44.7%), 156 HS (27.4%) and 159 TIA (27.9%). In the group of Russians, there were 277 IS (62.9%), 70 HS (15.9%) and 93 TIA (21.1%). In the group of patients of other ethnicities, there were 60 IS (61.2%), 10 HS (10.2%) and 28 TIA (28.6%). Thus, the number of patients with stroke (IS and HS) amounted to 828 cases, including Indigenous (n=411; 49.6%), Russians (n=347; 41.9%) and another ethnic patients (n=70; 8.5%).

A comparative analysis was made between the 2 most numerous ethnic groups of patients with stroke.

In the indigenous patients, 255 IS cases (62%) and 156 HS cases (38%) were diagnosed. Among patients of Russian ethnicity, 277 IS (79.8%) and 70 HS cases (20.2%) were diagnosed. When comparing the incidence of HS in different ethnic groups, we found that indigenous patients had more cases of HS than Russians (38% vs. 20.2%, p<0.05) (adjusted odds ratio=2.42; 95% CI: 1.72–3.41).

The average age of Indigenous patients with IS was 63.3±12.01 (range: 28–94 years), while in the IS Russians it was 63.7±11.8 (range: 24–88 years).

The average age of HS Indigenous patients was 55.05±13.4 (range: 16–83 years), while in the Russians it was 57.6±14.9 (range: 22–101 years).

Thus, the average age of patients with IS and HS had no significant differences by ethnicity (p=0.69; p=0.201, respectively, t-test).

In the next stage of this study, we randomized a group of patients with stroke of indigenous and Russian ethnicity living in the city of Yakutsk. In this group, there were 653 patients, including Indigenous (n=320; 49.1%) and Russians (n=333; 50.9%). There were 346 (52.9%) male and 307 female (47.1%). Four hundred and ninety cases of IS (75.04%) and 163 cases of HS (24.9%) were diagnosed in the group.

A demographic characteristic of the patients according to ethnicity and type of stroke is shown in Table I.

**Table I:** The demographic characteristics of patients with stroke, depending on ethnicity and type of stroke

	General group (n =653)	IS (n=490)	HS (n=163)
Indigenous (n, number of patients in this ethnic group in %)	320 (100%)	217 (67.8%)	103 (32.2%)
Male (n, number of patients of this type of stroke in %)	186 (58.1%)	136 (62.7%)	50 (48.5%)
Mean age (years)	62 [53; 72]	63 [55; 73]	58 [50; 66]
Russians (n, number of patients in this ethnic group in %)	333	273 (82%)	60 (18%)
Male (n, number of patients of this type of stroke in %)	160 (48%)	133 (48.7%)	27 (45%)
Mean age (years)	63 [54; 72]	64 [55; 73]	59 [53; 70]

There was no statistically significant difference in age between the 2 ethnic groups in the general group of patients with stroke (p=0.188, Mann–Whitney U-test).

When comparing the average age of IS indigenous patients (n=217) (63 [55; 73]; range: 28–94 years) and Russians (n=273) (64 [55; 73]; range: 24–88 years), no significant differences were found (p=0.957, Mann–Whitney U-test).

Among patients with HS, there were also no statistically significant differences in age by ethnicity (p=0.323, Mann–Whitney U-test). Thus, the average age of HS indigenous (n=103) was 58 [50; 66] (range: 16–83 years), while the average age of Russian HS patients (n=60) was 59 [53; 70] (range: 22–101 years).

Comparative analysis of the frequency of different types of stroke among ethnic groups revealed a significant difference. One hundred and three HS cases among indigenous patients (32.2%) and 60 HS cases among the Russians (18.02%) were registered. The number of IS cases was 217 (67. 8%) among the indigenous and 273 (82%) among the Russians. The ratio of HS: IS was 1:4.5 in the Russians and 1:2.6 in the Indigenous. Thus, the group of Yakutsk residents patients of native ethnicity had a greater number of HS compared with those of Russian ethnicity (Fisher’s test p=0.000001; adjusted odds ratio=2.16; 95% CI: 1.48–3.12) (32.2% vs. 18.02%, respectively).

## Discussion

Our study for the first time described the ethnic characteristics of stroke in Yakutia, based on data from Hospital registers and data neuroimaging, which was carried out on all examined patients. When comparing the HS morbidity in different ethnic groups, we found that indigenous patients had more cases of HS than the Russian patients. Mean age of IS and HS indigenous patients had no significant differences in comparison with the average patient age of Russian nationality.

In the previous surveys, the structural features of a stroke in Yakutia were elucidated. Thus, according to the territorial and population Stroke register held in Yakutsk in 2006, the trend toward a high frequency of HS among the indigenous population was specified, but the difference did not reach statistical significance (9). Perhaps, it was due to the fact that in their study, neuroimaging was performed in only 73.2% of patients with strokes, which could affect the diagnosis of the type of stroke. It was also shown that the proportion of HS in the general structure of stroke in Yakutia is higher in comparison with the indexes in Russia: the ratio of ischemic and HS was 2.2:1 (10). According to Skvortsova et al. data, intracerebral haemorrhage and subarachnoid haemorrhage comprise 14.1 and 3% of all strokes in Russia, respectively (11). Thus, the ratio of ischemic and HS is 4.8:1.

Our data also correspond to the information about the existence of ethnic differences in the incidence of intracerebral haemorrhage (12–16). Compared to the US white population, Alaska Natives have a greater mortality from stroke (from stroke and HS) at a young age (<45 years) (17).

In our study, the ethnic differences in the frequency of types of stroke, derived from data on the general base of the patients admitted from all parts of Yakutia, were confirmed with the database of patients hospitalized from Yakutsk city.

A limitation of our study is that the number of patients is relatively small, as the data from a 1-year period are used. In the future, it will be necessary to accumulate data on the basis of Hospital strokes. Moreover, it is necessary to create a database of patients with acute stroke admitted to other hospitals in Yakutia.

Established ethnic differences in the HS frequency are associated with factors that still remain unclear. It is necessary to investigate the risk factors for stroke in different ethnic groups in Yakutia, including the prevalence and severity of arterial hypertension. It is also necessary to examine the frequency of other causes of cerebral vessel’s ruptures, such as aneurysms and brain vascular malformations in different ethnic groups. Maybe there are structural features of the walls of cerebral blood vessels, such as the weakness of the connective tissue of cerebral vessels in the certain ethnic group. It is also necessary to investigate the features of social, climatic factors and changes in the way of life of different ethnic groups, which have occurred during the last decades in Yakutia.

## Conclusions

The data of our study suggest that among the patients with stroke in the indigenous population, the share of a haemorrhagic form is higher than in those of non-indigenous Caucasians. Simultaneously, the average age of patients, both IS and HS, displayed no significant differences by ethnicity. Further studies are needed to establish the causes of ethnic differences of stroke in Yakutia.
